# The effects of highly active antiretroviral therapy on the serum levels of pro-inflammatory and anti-inflammatory cytokines in HIV infected subjects

**DOI:** 10.1186/s12929-018-0490-9

**Published:** 2018-12-03

**Authors:** Faustina Nkechi Osuji, Charles Chinedu Onyenekwe, Joseph Ebere Ahaneku, Nkiruka Rose Ukibe

**Affiliations:** 1 Immaculate Heart Specialist Hospital, P.O.Box 29, Nkpor-Agu, Idemili-North L.G.A. Anambra State, Nnewi, Nigeria; 2 Department of Medical Laboratory Science, College of Health Sciences, Nnamdi Azikiwe University, Nnewi Campus, Anambra State Nigeria; 3 Department of Chemical Pathology, Nnamdi Azikiwe University Teaching Hospital, Nnewi, Anambra State Nigeria; 4 Department of Medical Laboratory Science, College of Health Sciences, Nnamdi Azikiwe University, Nnewi Campus, Anambra State Nigeria

**Keywords:** Cytokines, HIV, HAART

## Abstract

**Background:**

Cytokines play an important role in controlling the homeostasis of the immune system and infection with Human Immunodeficiency virus (HIV) leads to deregulated production of both pro- and anti-inflammatory cytokines. This study was designed to determine the effects of HIV and Highly Active Antiretroviral Therapy (HAART) on the levels of pro-and anti-inflammatory cytokines in HIV infected subjects.

**Method:**

A total of 50 HIV infected and 50 HIV seronegative control participants were recruited for the study. The HIV infected subjects were recruited before commencement of antiretroviral therapy and were followed up for 12 months. Blood samples were collected at 3 different points: before initiation of therapy, 6 months into therapy and 12 months into therapy. Serum cytokines were analyzed using ELISA method while CD4+ T cells and viral load counts were measured using standard laboratory methods.

**Result:**

The results showed that pro-inflammatory cytokines: Tumour necrosis factor-alpha (TNF-α), Interleukin-6 (IL-6) and anti-inflammatory cytokines Interleukin-4 (IL-4), Interleukin-10 (IL-10) and Transforming growth factor-beta (TGF-β) were significantly elevated in HIV infected subjects before commencement of therapy compared to 6 months and 12 months into therapy (*P* < 0.01) and compared to control participants (*P* < 0.01). TNF-α, TGF-beta remained significantly elevated even after 12 months of therapy compared to control participants (*P* < 0.01), while IL-4, IL-6, and IL-10 showed no significant difference compared to control participants after 12 months of therapy (*P* > 0.05). INF-γ was significantly reduced before commencement of therapy and after 12 months of therapy compared to control participants (*P* < 0.05) respectively.

**Conclusion:**

TNF-α and TGF-β remained significantly elevated even after 12 months of therapy, while IFN-γ remained significantly reduced after 12 months of therapy. Regulating these cytokines which were unresponsive to therapy could serve as a potential measure of therapy for HIV infected subjects. The positive effect of 12 months therapy on IL-4, IL-6 and IL-10 levels can be used to monitor disease prognosis during therapy especially in resource poor setting where regular viral load monitoring is unavailable.

## Introduction

Cytokines can be classified as pro and anti-inflammatory and are considered important initiators and mediators of inflammation [1]. Infection with HIV results in the production of cytokines by infected cells and cells of the immune system. Such cytokines regulate the immune function and affect viral replication [2]. Changes in cytokine levels in HIV infected individuals can affect the function of the immune system and have the potential to directly impact the course of HIV disease by enhancing or suppressing HIV replication [3]. It has been suggested that an imbalance in cytokine production is responsible in part for the immune deregulation characteristic of progression of HIV to AIDS [2].

Both innate and adaptive immune responses are raised during HIV infection. CD4+ T helper cells play a vital role in the immune system by secreting cytokines, which regulate the immune response. Cytokines are secreted by T cells when an intracellular infection is detected as in the case of HIV infection. HIV is known to infect T cells that have CD4+ receptors present on their surface. These cells, among others in the immune system, secrete cytokines. The level of cytokines present in the plasma becomes an indicator of the nature of the immune response. Therefore those cytokines secreted by CD4+ T cells during HIV infection are expected to be low. Th1 CD4 + subset produce IL-2 and IFN-γ. IFN-γ acts by activating macrophages and is essential for eliminating intracellular pathogens [4]. Most of these cytokines have been used as hallmarks of disease progression as well as assessment of patient’s response to antiretroviral treatment [5]. During the course of HIV-1 infection, secretion of T-helper type 1 (Th 1) cytokines such as interleukin (IL-2) and interferon gamma (IFN-γ) are generally decreased, whereas production of T helper 2 (Th 2) cytokines IL-4, IL-10, proinflammatory cytokines (IL-1, IL-6, IL-8) and tumour necrosis factor TNF-α are increased [6]. Such abnormal cytokine production contributes to the pathogenesis of the disease by impairing cell-mediated immunity [7]. It has been previously documented that HIV replication is the result of a balance between the effects of pro-inflammatory cytokines that increase viral replication and those of anti-inflammatory cytokines and chemokines that inhibit viral replication [8].

HAART includes the combination of three or more different types of highly effective anti-HIV drugs including nucleotide reverse transcriptase inhibitors (NRTIs), non-nucleoside reverse transcriptase inhibitor (NNRTIs) and protease inhibitors (PIs). Successful HAART suppresses viral replication at different stages of HIV life cycle, improves immunological status of HIV infected individuals and delays drug resistance. Overall the use of HAART results in marked improvement in the prognosis of HIV disease [9]. With the growing understanding of their roles during infections and disease progression, cytokines including IFN-γ, IL-10 and tumor necrosis factor alpha (TNF-α) have been assayed in plasma to assess the efficacy of antiretroviral therapy during HIV infection [10, 11]. For example, HAART markedly increases plasma IFN-γ levels [12, 13], and considerably lowers IL-10 systemic levels during HIV infection [11]. Hence, these actions co-operatively show that antiretroviral treatment markedly influences systemic cytokine levels.

The prognostic and pathophysiologic role of pro- and anti-inflammatory cytokines in HIV infection remains unclear, and information regarding cytokine profiles that are characteristic of either clinical improvement of disease or progression is unclear especially in developing countries. The effectiveness of HAART could be affected by the balance between pro- and anti-inflammatory cytokines which are involved in regulating immune response to HIV. Evaluating the levels of these cytokines may give a picture of the rate of viral replication as well as damage or restoration of immune system especially during HAART.

## Materials and methods

### Study population

A total of one hundred subjects were randomly recruited for this study, which consists of 50 HIV infected individuals and 50 HIV negative control individuals. The recruitment and study was carried out at Nnamdi Azikiwe University Teaching Hospital Nnewi. The study subjects were followed up for 12 months after commencement of HAART. Blood samples were collected at 3 different points: before initiation of HAART, 6 months into HAART and 12 months into HAART.

#### A. HIV positive subjects

The HIV infected subjects were aged 20–69 years (39 ± 10) years and were recruited before commencement of HAART. (Male =19, Female =31).

#### Before commencement of HAART

These were individuals confirmed for HIV-1/2 seropositivity. They were eligible for first line HAART. These individuals were started on HAART after sample collection.

#### 6 months into HAART

These were the same subjects recruited before commencement HAART but who have taken HAART for 6 months. Blood samples were collected from them exactly after 6 months intake of HAART.

#### 12 months into HAART

These were the same participants recruited before commencement of HAART but who have taken HAART for 12 months. Blood samples were collected from them exactly after 12 months intake of HAART.

#### B. Control subjects

The control subjects were HIV seronegative aged (21–56) (35 ± 10 years). (Male = 21, Female =29).

### Inclusion and exclusion criteria

Individuals were eligible if ≥18 years and eligible for HAART initiation. Individuals with pre-existing symptoms of AIDS according to WHO criteria at the base line visit were excluded from the programme. In addition those with previous exposure to HAART, and those with a history of diabetes mellitus, active opportunistic infections, inflammatory conditions, diarrhoea or pregnancy were excluded. The control subjects were apparently healthy HIV seronegative individuals and were eligible if ≥18 years.

### Study design

The study design was a longitudinal cohort study in which blood samples were collected from study subjects before initiation of HAART, 6 months into HAART and 12 months into HAART.

### Sample collection

Ten millilitres of fasting blood were drawn by venipuncture from all subjects in the study. The blood samples were aliquoted appropriately into k_3_-EDTA for CD4+ T cell, and Viral load and serum separator tubes (SST) for obtaining clear serum for the determination of cytokines (IL-4, IL-6, IL-10, TNF-α, INF-γ and TGF-β. The sera were separated and stored at ≤ -20 °C until analyses.

## Methods

### Determination of CD4+ T-cells count by CyFlow SL green

20 μl of whole blood in EDTA anti-coagulant was dispensed into a Partec test tube and 20 μl of CD4 PE antibody was added. The reaction mixture was incubated in the dark for 15 min. After incubation, 800 μl of the already prepared diluted buffer (Xn 0.09% NaN_3_) was added to each reaction tube and vortexed. The partec tubes containing these reactions were plugged in position in the Cyflow SL Green (Partec Germany), which has already been connected to flow max software, CD4 count template data file and CD4 count instrument. The test was run on the Cyflow for 90 s. The results were displayed as histogram and printed. The CD4+ T-Cell count was read off the histogram correcting for the dilution factor.

### Viral load measurement

HIV-1 RNA quantitation was by Amplicor HIV-1 Monitor (Roche Diagnostics Corporation; Branchburg, New Jersey, USA). The UltraSensitive procedure, performed according to the manufacturer’s recommendations, has a measuring range of 20–750,000 RNA copies/mL. During the specimen preparation procedure, the HIV 1/2 viral particles in plasma are concentrated by high speed centrifugation, followed by lysis of the virus particles with a chaotropic agent containing Tris-HCl buffer, 68% Guanidine thiocyanate, 3% Dithiothreitol and < 1% Glycogen. This is followed by precipitation of the HIV RNA with alcohol. A known number of quantitation standard RNA molecules are introduced into each specimen with the lysis reagent. The HIV Quantitation Standard is carried through the specimen preparation, reverse transcriptase, amplification and detection steps and is used for the quantitation of HIV RNA in each specimen.

### Cytokine measurement

IL-6, IL-4, IL-10, TNF-α, INF-γ, and TGF-β cytokines were determined by ELISA with Human Quantkine® Elisa Kits (R&D Systems, USA).

The method employs the quantitative sandwich enzyme immunoassay technique. A monoclonal antibody specific for human IL-6, IL-4, IL-10, TNF-α, INF-γ, and TGF-β has been coated onto a microplate for each cytokine respectively. Subsequently, 200 μl of test serum, positive and negative controls was added to the microplates and were incubated for 2 h at room temperature. Four washes were carried out with detergent solution containing buffered surfactant with preservatives. 200 μl of conjugate was added and incubated for 1 h at room temperature. The washing step was repeated and 200 μl of substrate solution was added and incubated in the dark for 20 min. The reaction was stopped with 2 N sulphuric acid. Results were evaluated by optical density (OD) on a Mindray microplate reader at 450 nm. Serum cytokines were calculated from a standard curve.

### Statistical analysis

The results were presented as mean ± standard deviation. Differences between the results of the control subjects and those of HIV positive subjects before commencement of HAART, 6 months and 12 months into HAART were analyzed using student’s *t* test and ANOVA. Pearson correlation was employed in analyzing the relationship between viral load count and the cytokines under study. SPSS statistical package version 22 was used in data analysis. Significant levels were considered at *P* < 0.05.

### The antiretroviral drug combination for the HIV subjects

- Tenofovir 300 mg, Lamivudine 300 mg, Efavirenz 600 mg (TENOLAM E). This is a ‘first line’ drugs combined in a single pill.
- Dosage and Administration: Adults and adolescents weighing ≥30 kg: 1 tablet once daily in the evening. There was no adverse drug reactions reported by the subjects during therapy and there was no interruption of therapy by the subjects. The drug combination, dosage and administration are according to WHO preferred initial regimen for adults and adolescents as of June 2013, which include: tenofovir + lamivudine + efavirenz (i.e 2 NRTIs and a NNRTI). (WHO, 2013). This first line drugs can only be changed for a patient experiencing adverse drug reaction or in cases of drug resistant which is indicated by high viral load count after 6 months of therapy. None of the subjects showed adverse drug reaction or drug resistant.

## Results

Table 1 shows significant differences in serum levels of anti-inflammatory cytokines IL-4 (pg/ml), IL-10 (pg/ml) and TGF-β before commencement of HAART, 6 months into HAART and 1 year into HAART (*P* < 0.01). Group comparison shows that these anti-inflammatory cytokines measured were significantly higher before HAART initiation compared to control participants (*P* < 0.01) and compared to six months into therapy (*P* < 0.05). IL-4 and IL-10 showed no significant difference after 1 year of HAART compared to levels in control participants (*P* > 0.05), while TGF-β remained significantly high in HIV infected subjects after 1 year of HAART compared to control participants (*P* < 0.05).

**Table 1:** Serum anti-inflammatory cytokine Levels and CRP (ng/ml) amongst HIV infected subjects before commencement of HAART, 6 months into HAART and 1 year into HAART (±SD)

Group	IL-4 (Pg/ml)	IL-10 (Pg/ml),	TGF-β (Pg/ml),
Before commencement of HAART {a} (*n* = 50)	26.67 ± 4.39	31.57 ± 6.35	102.77 ± 30.53
6 Months into HAART{b} (*n* = 50)	7.98 ± 6.93	18.47 ± 5.29	85.03 ± 28.33
1 Year into HAART{c} (*n* = 50)	4.42 ± 2.11	7.05 ± 2.43	61.24 ± 22.08
Control {d} (*n* = 50)	3.68 ± 1.59	4.66 ± 2.12	39.20 ± 14.39
*F* Value	15.93	30.47	99.70
*P* Value	0.02	0.00	0.00
{a} Vs {b}	*P* < 0.05	*P* < 0.05	*P* < 0.05
{a} Vs {c}	*P* < 0.05	*P* < 0.05	*P* < 0.05
{a} Vs {d}	*P* < 0.05	*P* < 0.05	*P* < 0.05
{b} Vs {c}	*P* < 0.05	*P* < 0.05	*P* < 0.05
{b} Vs {d}	*P* < 0.05	*P* < 0.05	*P* < 0.05
{c} Vs {d}	*P* > 0.05	*P* > 0.05	*P* < 0.05

*IL-4* Interleukin 4, *IL-10* Interleukin 10, *TGF-β* Tissue Growth Factor Beta, *HAART* Highly Active Antiretroviral Therapy, *Vs* Versus

Table 2 shows significant differences observed in mean serum levels of pro-inflammatory cytokines IL-6 (pg/ml), TNF-α (pg/ml) and INF-γ (pg/ml) before commencement of HAART, 6 months into HAART and 1 year into HAART (*P* < 0.01). Within group comparison shows that IL-6 and TGF-β were significantly high before HAART initiation compared to levels in control participants (*P* < 0.01) and compared to 6 months into HAART (*P* < 0.05). TNF-α remained significantly high after 1 year of HAART compared to control participants *P* < 0.01), while IL-6 shows no significant difference when compared to control participants after 1 year of therapy (*P* > 0.05). INF-γ was significantly low before HAART initiation (*P* < 0.01) and after 1 year of therapy compared to control participants (*P* < 0.05).

**Table 2:** Serum pro-inflammatory cytokine Levels amongst HIV infected subjects before commencement of HAART, 6 months into HAART and 1 year into HAART (±SD)

Group	IL-6 (Pg/ml)	INF-γ (Pg/ml)	TNF-α (Pg/ml)
Before commencement of HAART {a} (*n* = 50)	18.34 ± 5.83	0.78 ± 0.06	41.32 ± 12.99
6 Months into HAART{b} (*n* = 50)	10.43 ± 4.98	2.25 ± 0.91	29.32 ± 10.51
1 Year into HAART{c} (*n* = 50)	7.05 ± 3.43	2.64 ± 0.96	15.98 ± 4.81
Control {d} (*n* = 50)	4.62 ± 1.04	4.89 ± 0.23	9.40 ± 3.52
*F* Value	60.90	11.37	75.62
*P* Value	0.00	0.02	0.00
{a} Vs {b}	*P* < 0.05	*P* < 0.05	*P* < 0.05
{a} Vs {c}	*P* < 0.05	*P* < 0.05	*P* < 0.05
{a} Vs {d}	*P* < 0.05	*P* < 0.05	*P* < 0.05
{b} Vs {c}	*P* < 0.05	*P* < 0.05	*P* < 0.05
{b} Vs {d}	*P* > 0.05	*P* < 0.05	*P* < 0.05
{c} Vs {d}	*P* > 0.05	*P* < 0.05	*P* < 0.05

*IL-6* Interleukin 6, *INF-γ* Interferon Gamma, *TNF-α* Tumour Necrosis Factor alpha, *Vs* Versus, *HAART* Highly Active Antiretroviral Therapy, *n* number of participants

Table 3 shows that the blood concentration of CD4+ T Cell (/μl) and viral load (copies/ml) in HIV infected subjects before commencement of HAART, 6 months into HAART, 1 year into HAART and that of control subjects were significantly different *P* < 0.01. Between group comparison showed that the CD4+ T cell count was significantly lower before commencement of HAART compared to 6 months into HAART and 1 year into HAART (*P* < 0.05) respectively. There was also a significant lower CD4 T cell count in 1 year into HAART compared with levels in control subjects (*P* < 0.05). After one year of HAART most participants showed undetectable viral load.

**Table 3:** CD4 + T cell count (/μl) and viral load (Log_10_ copies/mL) in HIV infected subjects before commencement of HAART, 6 months and 1 year into HAART (±SD)

Group	CD4+ T cells /μl	Viral Load(Log_10_ copies /ml)
Before commencement of HAART {a} (*n* = 50)	152 ± 67	5.45 ± 0.37
6 Months into HAART {b} (*n* = 50)	378 ± 162	2 .70 ± 0.63
1 Year into HAART{c} (*n* = 50)	522 ± 160	0.18 ± 0.04
Control {d} (*n* = 50)	900 ± 245	–
*F* Value	67.73	33.09
*P* Value	0.00	0.00
{a} Vs {b}	*P* < 0.05	*P* < 0.05
{a} Vs {c}	*P* < 0.05	*P* < 0.05
{a} Vs {d}	*P* < 0.05	–
{b} Vs {c}	*P* < 0.05	*P* < 0.05
{b} Vs {d}	*P* < 0.05	–
{c} Vs {d}	*P* < 0.05	–

*Vs* Versus, *HAART* Highly Active Antiretroviral Therapy, *n* number of participants

Table 4 shows that in HIV infected participants before initiation of HAART there was a significant positive correlation observed between viral load and IL-6 (*r* = 0.62, *P* < 0.01), between viral load and TNF-α (*r* = 0.44, *P* < 0.05), between viral load and IL-4 (*r* = 0.34, P < 0.05), and between viral load and TGF-β (*r* = 0.57, P < 0.01) in HIV infected subjects before commencement of HAART. There was also a negative correlation between viral load and INF-γ (*r* = − 0.43, *P* < 0.05) and between viral load and CD4+ T cells (*r* = 0.72, P < 0.01) in HIV infected subjects before commencement of HAART, but there was no significant correlation between viral load and IL-10 (*r* = 0.12, *P* > 0.05) in HIV infected subjects before commencement of HAART.

**Table 4:** Correlation between viral load (Log_10_ copies/ml), CD4 + T cells (/μl), and interleukins in HIV infected subjects before commencement of HAART

parameters	n	r	*P* value
Viral load Vs IL-4	50	0.34	< 0.05
Viral Load Vs IL-6	50	0.62	< 0.05
Viral Load Vs IL-10	50	0.02	> 0.05
Viral Load Vs INF-γ	50	−0.43	< 0.05
Viral Load Vs TNF-α	50	0.44	< 0.05
Viral Load Vs TGF-β	50	0.57	< 0.05
Viral load and CD4+ T cells	50	0.72	< 0.01
CD4+ T cells and IL-4	50	−0.34	< 0.05
CD4+ T cells and INF-γ	50	0.42	< 0.05
CD4+ T cells and TNF-α	50	−0.42	< 0.05
CD4+ T cells and TGF-β	0	−0.66	< 0.01

Correlation was analyzed by Pearson’s correlation

*Vs* Versus Vs, *HAART* Highly Active Antiretroviral Therapy, *n* number of participants, *IL-4* Interleukin 4, *IL-10* Interleukin 10, *TGF-β* Tissue Growth Factor Beta

*IL-6* Interleukin 6, *INF-γ* Interferon Gamma, *TNF-α* Tumour Necrosis Factor alpha

Table 5 shows that there was no significant correlation observed between viral load and IL-4, IL-10, IL-6, INF-γ, and TNF-α in HIV infected subjects after 1 year into HAART, but there was a significant correlation between viral load and TGF-β (*r* = 0.39, *P* < 0.05) viral load and CD4+ T cells (*r* = 0.33, *P* < 0.05) respectively in HIV infected subjects after 1 year intake of HAART.

**Table 5:** Correlation between Viral load (Log_10_ copies/ml), CD4 + T cells (/μl), and interleukins in 1 year into HAART subjects

Parameters	n	r	*P* value
Viral load Vs IL-4	50	0.23	*P* > 0.05
Viral Load Vs IL-6	50	0.09	*P* > 0.05
Viral Load Vs IL-10	50	0.26	*P* > 0.05
Viral Load Vs INF-γ	50	−0.16	*P* > 0.05
Viral Load Vs TNF-α	50	−0.18	*P* > 0.05
Viral Load Vs TGB-β	50	0.39	*P* < 0.05
Viral load and CD4+ T cells		0.42	*P* < 0.05

Correlation was analyzed by Pearson’s correlation

*Vs* Versus Vs, *HAART* Highly Active Antiretroviral Therapy, *n* number of participants, *IL-4* Interleukin 4, *IL-10* Interleukin 10, *TGF-β* Tissue Growth Factor Beta

*IL-6* Interleukin 6, *INF-γ* Interferon Gamma, *TNF-α* Tumour Necrosis Factor alpha

## Discussion

This study was carried out to determine the effect of antiretroviral therapy on the levels of pro- and anti-inflammatory cytokines in HIV infected subjects before commencement of HAART and through 1 year of therapy. The longitudinal assessment of the serum cytokines measured showed that TNF-α, IL-6 (Pro-inflammatory cytokines) and IL-10, IL-4 and TGF-β (anti-inflammatory cytokines) were significantly high in HIV infected subjects. The increase was more marked before commencement of HAART which gradually became lowered by 1 year of HAART though TNF-α and TGF-β failed to return to normal levels when compared with control subjects.

HIV infection is accompanied by a robust plasma pro-inflammatory and anti-inflammatory cytokine response in HAART naive individuals. The pro-inflammatory cytokines seen to be high in this study were probably produced by monocytes and macrophages to initiate inflammation which is essential for the development of innate immunity while the anti-inflammatory cytokines seen to be increased were stimulated probably to deactivate the activities of the pro-inflammatory cytokines [14]. The above observation is supported by the fact that there was a strong significant correlation between viral load and the pro-inflammatory cytokines and anti-inflammatory cytokines before commencement of HAART suggesting that these cytokines were induced in response to HIV replication in the absence of HAART.

Interleukin-4 a definer of Th-2 subset exerting dominant anti proliferative effects is identified among the critical cytokines promoting HIV immunopathology [15]. However, with HAART commencement, expressed levels of this cytokine gradually decline to those comparable with healthy controls [16]. This may be linked to marked reduction in HIV viral load with subsequent suppression of inflammatory episodes resulting from combined antiretroviral therapy. Also the pro-inflammatory mediator TNF-α seen to be elevated in the study was reported to be implicated in HIV pathogenesis through induction of virus transcription-activating factors [17]. Previous studies conducted by Meira et al. [18] documented lower TNF-α, IL-4 and IL-10 serum level among HIV-1 mono-infected ART-experienced compared to their ART-naive counterparts. Similarly, elevated levels of TNF-α have been reported in mononuclear phagocytes during HIV, and which significantly decline during HAART [19]. A heightened level of these three cytokines hastens HIV replication and has been associated with poor HIV outcomes [20, 21]. Thus the observed findings above delineate HAART to induce immunological recuperation while down-regulating cytokine associated inflammation and immune activation.

The study also shows that serum IFN-γ though a pro-inflammatory cytokine was significantly lower in HIV infected subjects before commencement of HAART and remained significantly low after 1 year of HAART. Various studies describe a decrease in IFN-γ levels during acute HIV-1 infection, which markedly increases during HAART [12, 13]. This may be related to immune restoration following viral suppression. Contrastingly, more recent studies document significant decline of plasma IFN-γ levels with HAART administration among previously untreated HIV-1 infected subjects [22], this may be related to down-regulation of inflammation and immune activation by antiretroviral drug intervention. Collectively, these contradictory findings may be attributed to distinct mechanisms of immune activation that are differentially affected by antiretroviral therapy.

Successful antiretroviral therapy has previously been reported to lower plasma IL-10 level [11], which is paralleled by a reduction in viral load among HIV-1 infected individuals [23]. This indicates that high HIV viral load may be the main driver of high plasma IL-10 levels, which significantly reduce upon effective treatment with antiretroviral therapy as observed in the study. Furthermore a lack of decline in plasma IL-10 levels following HAART administration has been associated with virologic treatment failure [24], which further reinforces the above argument.

A study conducted by Gay et al., [25] found minimal effects of HAART on the levels of cytokines after 16 to 24 weeks of treatment. Some other reports did not observe any influence of HAART on levels of IL-6 [26, 27] while other reports [23, 28] observed a decrease of IL-6 and TNF-α after initiating HAART. Sachdeva et al., [29], found similar results in serum levels of IL-6, TNF-α, IFN-γ and IL-12, which decreased after 6 months of HAART, but some of these values were still higher than those of control individuals. Still, in a study by Haissman et al. [28], 4 months after HAART initiation there was a significant reduction, mainly to the TNF-α and IL-6 in individuals with elevated CD4+ T cells. This divergent of data in cytokine studies can be attributed to the various factors responsible for the incomplete restoration of the immune function despite HAART, as late initiation of therapy, low CD4 + nadir, slow immune reconstitution and poor therapeutic adherence could be responsible.

While HAART typically decreases both immune activation and inflammatory markers, recent studies showed that these markers remain elevated in many HIV infected patients (Abino et al. 2016). Continued immune activation and inflammation in HIV infected on HAART is likely the result of several mechanisms like persistent HIV replication on tissue sites such as lymph nodes, the gastrointestinal tract and increased levels of inflammatory lipids [30]. The importance of co-infections on chronic immune activation in HAART treated HIV infected should not be overlooked as co-infections with hepatitis B virus, Hepatitis C virus or cytomegalovirus are common with HIV infection and have been linked with heightened CD4 + T and CD8+ T cell activation [9]. Non-AIDS defining co-morbidities like cardiovascular diseases, insulin resistance and type II diabetes, osteoporosis and cancer also result in persistent immune activation and inflammation, though subjects with these co-morbidities were excluded from the research. Aging can also result in persistent immune activation and inflammation [31], as subjects in the study were aged between 20 and 69 years.

The CD4+ T cell count is an important biomarker for HIV progression; our results showed that CD4 + T cells count were significantly lower in HIV infected subjects. This may have contributed to the altered cytokine profiles of the HIV positive subjects. A significant recovery of CD4+ T cell count was demonstrated 6 months and 12 months after starting HAART. Hainaut et al., [32], had earlier reported a significant increase in CD4+ T cells count between 3 months to 6 months of HAART in their study. This clearly demonstrated general immune reconstitution after commencement of HAART. Various studies have documented that HAART leads to immune reconstitution and viral suppression during HIV infection [23, 24].

## Conclusions and future perspectives

HIV infection is associated with chronic immune activation and dysfunctional cytokine production. HAART induced a significant gradual increase in CD4+ T cell count, decrease in viral load, IL-4, IL-6 and IL-10 close to those in HIV uninfected subjects. While INF-γ, TGF-β and TNF-α was not completely responsive to HARRT after 1 year of therapy. These findings may indicate potential usefulness of IL-4, and IL-10 which are anti-inflammatory cytokines and IL-6 a pro-inflammatory cytokine as a marker of HIV disease progression and a marker of successful HAART in combination with CD4+ T cell count and viral load test. Regulating the release of those cytokines which were unresponsive to HAART may also serve as a potential measure of therapy for HIV infected individuals. On the whole, more investigations on anti- inflammatory and pro-inflammatory cytokines are required in order to potentiate their utility as predictors of HIV disease progression and response to treatment among HIV infected subjects. Measurement of these cytokines could be extended beyond one year of therapy so as to arrive at a better conclusion on the effects of HAART on cytokine levels.

## Abbreviations

HAART: Highly Active Antiretroviral Therapy
HIV: Human Immunodeficiency Virus;
IFN-γ: Interferon gamma
IL-10: Interleukin-10
IL-4: Interleukin-4
IL-6: Interleukin-6
NK cells: Natural Killer Cells
TGF-β: Tissue Growth Factor beta
Th1: Type 1 Helper T-Lymphocytes
TNF-α: Tumor Necrosis Factor Alpha
WHO: World Health Organisation
